# Hematoma evacuation based on active strategies versus conservative treatment in the management of moderate basal ganglia hemorrhage: A retrospective study

**DOI:** 10.1515/tnsci-2022-0292

**Published:** 2023-07-29

**Authors:** Weihua Zhang, Jian Zhang, Gaoming Huang, Kaichuang Yang

**Affiliations:** Center for Rehabilitation Medicine, Department of Neurosurgery, Zhejiang Provincial People’s Hospital (Affiliated People’s Hospital), Hangzhou Medical College, Hangzhou, Zhejiang, China; Department of Neurosurgery, The First People’s Hospital of Aksu Region, Aksu, Xinjiang, China; Center for Rehabilitation Medicine, Department of Neurosurgery, Zhejiang Provincial People’s Hospital (Affiliated People’s Hospital), Hangzhou Medical College, Hangzhou, Zhejiang, China

**Keywords:** basal ganglia hematoma, conservative treatment, craniotomy, ultra-early, transsylvian-transinsular approach.

## Abstract

**Objective:**

The internal capsule of the basal ganglia is vulnerable to direct pressure from the hematoma and to secondary damage from toxic products of hemorrhage. Our study evaluated the risk and benefits of active strategies including ultra-early surgery and hematoma evacuation through a transsylvian-transinsular approach for moderate basal ganglia hemorrhage.

**Methods:**

We retrospectively collected patients with moderate basal ganglia hemorrhage in two hospitals. The conservative group contained 51 patients who had the best medical treatment, and the surgery group contained 36 patients who were treated with hematoma evacuation through a transsylvian-transinsular approach within 6 h from ictus. Motor function of upper and lower limbs recorded with the motor sub-score of NIHSS (m-NIHSS) at the baseline, 7 days, 30 days, and 90 days, the modified Rankin Scale (mRS), and Barthel Index (BI) scores at 30 and 90 days were compared between the two groups. Good recovery was defined as an m-NIHSS of 0–2 and poor recovery as 3–4. Favorable prognosis was defined as an mRS of 0–3 and unfavorable prognosis as 4–5.

**Results:**

The mean time from ictus to surgery was 250.3 ± 57.3 min. The good recovery proportions of upper and lower limbs in the surgery group were significantly higher than that in the conservative group (*p* < 0.05) at 7 days after hemorrhage. The good recovery proportion of upper limbs was significantly higher in the surgery group than in the conservative group (*p* < 0.05) at 3 months after hemorrhage. Living ability using BI scores was significantly higher in the surgery group than the conservative group (*p* < 0.05) at 3 months after hemorrhage. The favorable prognosis proportion had no statistically significant difference between the two groups at 3 months after hemorrhage.

**Conclusions:**

Ultra-early hematoma evacuation through a transsylvian-transinsular approach are active strategies for moderate basal ganglia hemorrhage and have potential advantages in improving motor function recovery and daily living. The postoperative rebleeding rate does not increase simultaneously.

## 1 Introduction

Moderate basal ganglia hematoma seldom causes brain hernia but mostly leads to neurological dysfunction. The internal capsule is vulnerable to direct pressure from the hematoma and secondary damage from toxic products of hemorrhage. To interrupt the chain reaction of hematoma, active strategies including early decompression of the internal capsule should be considered reasonable. To address better motor function restoration, the authors figured out the positive measurements from the multidimensional prognostic factors. First, the authors assigned ultra-early (<6 h from ictus) operative time window a high priority to obtain prompt decompression of the internal capsule. Second, craniotomy was employed as a surgical intervention in this retrospective study according to “2020 Chinese Expert Consensus on Endoscopic Surgery for Hypertensive Cerebral Hemorrhage,” which proposed that endoscopic intervention was recommended to be conducted after 6 h from ictus [1]. Third, the transsylvian-transinsular approach for basal ganglia hemorrhage evacuation was proved to have less operative disturbance than conventional craniotomy and purposely applied in this study [2,3,4,5]. Hence, we screened the patients who were in accordance with the mentioned measurements from those with moderate basal ganglia hematoma and divided them into the surgery group. The motor function recovery was then evaluated between the surgery group and the conservative group.

## 2 Materials and methods

### 2.1 Patient selection

From January 2019 to December 2021, consecutive patients with basal ganglia hemorrhage admitted to the department of neurosurgery in Zhejiang Provincial People’s Hospital and The First People’s Hospital of Aksu Region were retrospectively selected as the research subjects. The former hospital contributed 31 surgical cases and 22 conservative cases, while the latter finished the rest. The diagnosis of ICH was confirmed by computerized tomography (CT) scan of the head. The formula height × width × length × 0.5 according to the CT scan was used to calculate the hematoma volume [5]. Baseline parameters were defined as the last record within 6 h from ictus for the conservative group, whereas the last examination preoperatively for the surgery group. With consent from the patient and/or the patient’s family members, a surgery would be performed. Four neurosurgeons listed as authors performed all the surgeries. Conservative management was continued for all patients who refused invasive treatment and the best medical management prescribed by the 2015 AHA and Chinese guidelines was followed. Patients were divided into a surgery group and a conservative group according to the treatment they received. The motor function of the upper and lower limbs was documented separately using the motor sub-score of the NIHSS scale, which was recorded as m-NIHSS and ranged from 0 to 4 at the baseline, 7 days, 30 days, and 90 days after hemorrhage.

Inclusion criteria were as follows: (1) 40–75 years old; (2) hematoma volume between 25 and 40 ml, located from the frontotemporal cortex surface >1.5 cm, not entered into the ventricle; (3) time interval from ictus to surgery <6 h was used to screen for the surgery group; (4) m-NIHSS (motor function sub-score of NIHSS) of affected limbs = 3 or 4; and (5) preoperative Glasgow Coma Scale (GCS) >8 points. Exclusion criteria were as follows: (1) hemorrhage associated with aneurysm, cerebrovascular malformation, moyamoya disease, or trauma, preoperative CTA and intraoperative screening were performed to rule out these disease entities; (2) patients with severe organ dysfunction and abnormal coagulation function; (3) patients with previous cerebral hemorrhage or cerebral infarction; and (4) other postoperative diseases affect the rehabilitation of patients.

### 2.2 Patient demographic and clinical characteristics

All patients had no in-hospital death. Sixty patients had conservative treatment. Seven cases were lost to follow-up, and 2 cases died outside hospital during follow-up (1 case of heart attack and 1 case of intracranial bleeding). Finally, 51 cases were included in the analysis of the conservative group. Forty-three patients underwent surgical treatments, 6 patients were lost to follow-up, and 1 died of unknown causes outside the hospital. Finally, 36 patients were included in the surgery group. The clinical characteristics of the two groups are recorded in Table 1.

**Table 1:** Patient demographic and clinical characteristics

	Conversation group (51)	Surgery group (36)	*p* value
Age (years)	61.2 ± 9.0	60.4 ± 6.9	0.658
Male sex	36 (70.6%)	23 (63.9%)	0.510
Hematoma location left	20 (39.2%)	19 (52.8%)	0.210
Hematoma volume (ml)	33.8 ± 3.7	34.8 ± 1.4	0.232
Median (IQR, range)	33.6 (30.4, 37.1; 25.6–39.8)	35.5 (32.7, 38.3; 25.5–39.9)	
GCS at admission	11.5 ± 1.9	11.7 ± 2.0	0.629
**Hemorrhagic risk factors**			
Hypertension	31 (60.8%)	22 (61.1%)	0.975
Coronary heart disease	24 (47.1%)	19 (52.8%)	0.599
Diabetes mellitus	25 (49.0%)	19 (52.8%)	0.730
Pulmonary disease	33 (64.7%)	24 (66.7%)	0.850
Smoking	24 (47.1%)	17 (47.2%)	0.988
Habitual alcohol intake	36 (70.6%)	23 (63.9%)	0.510
**Surgery variables**			
Time from ictus to surgery (min)	NA	250.3 ± 57.3	NA
Median (IQR, range)	NA	241 (202, 301; 160–356)	NA
Hematoma clearance (%)	NA	89.7 ± 2.3	NA

Values were expressed as mean ± SD, median (interquartile range), or counts (%). SD: standard deviation; IQR: interquartile range; GCS: Glasgow Coma Scale. NA: not applicable. *p*-value using Chi-squared tests or Student’s *t*-tests.

### 2.3 Clinical treatments

In the two centers, all patients in the emergency room, ICU, and neurology ward received the best medical treatment complied with the Chinese guidelines and early rehabilitation (including physical exercises and acupuncture) according to the ERAS (Enhanced Recovery After Surgery) principle [1,6]. If necessary, they were individualized with particular treatments. In the surgery group, hematoma was removed through a transsylvian-transinsular approach under a microscope. The operation time was less than 3 h. Postoperatively, the patients were intubated and ventilated for a mean period of 1.8 days and kept in the intensive care unit for 2.6 days (mean).

### 2.4 Follow-up monitoring

**Figure 1:**
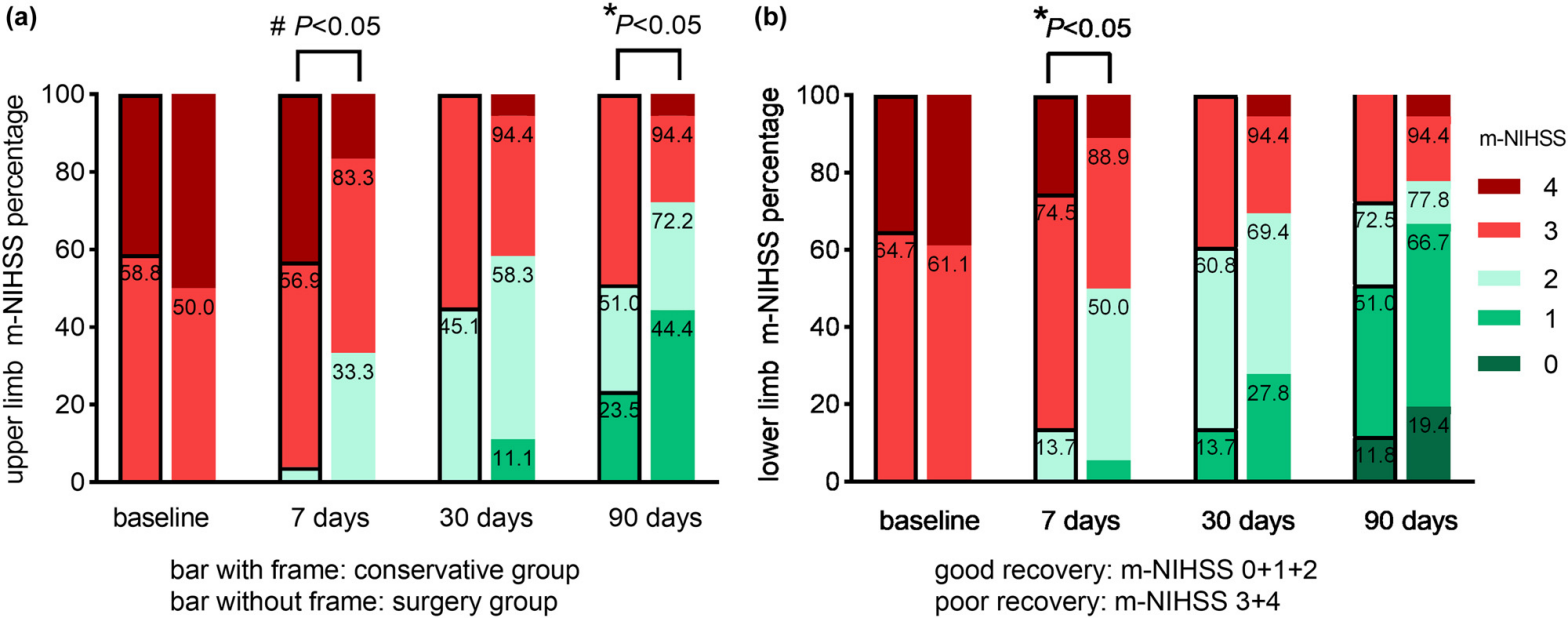
Comparison of the percentage of good recovery and poor recovery with the conservative group (bar with frame) and surgery group (bar without frame) at different time points during follow-up. Good recovery was defined as an m-NIHSS of 0 + 1 + 2 and poor recovery as 3–4. (a) Upper limb and (b) lower limb; ^#^continuous corrected *χ* ^2^ test; *χ ^2^ test.

The m-NIHSS of upper and lower limbs was recorded at the baseline, 7 days, 30 days, and 90 days after hemorrhage [7], while the modified Rankin Scale (mRS) and Barthel Index (BI) scores were recorded at 30 and 90 days during follow-up (Figure 1, Table 2). Follow-up information was obtained through electronic medical records, outpatient follow-up, telephone, WeChat messages, and video phones. Good recovery was defined as an m-NIHSS of 0–2 and poor recovery as 3–4. Favorable prognosis was defined as an mRS of 0–3 and unfavorable prognosis as 4–5.

**Table 2:** Comparison of BI and mRS between the two groups

	Conservative group (51)	Surgery group (36)	*p* value
BI at 30 days	48.2 ± 13.6	52.1 ± 18.8	0.270
BI at 90 days	58.0 ± 13.1	64.9 ± 17.0	0.037*
mRS at 30 days (good recovery)	26 (51.0%)	23 (63.9%)	0.232
mRS at 90 days (good recovery)	34 (66.7%)	28 (77.8%)	0.259

mRS: the modified Rankin Scale; BI: barthel index. *The 90-day BI score of the surgical group was better than that of the conservative group (*p* < 0.05).

### 2.5 Statistical analysis

SPSS software (Version 26; SPSS Inc, Chicago, IL, USA) was used to analyze the data. The data were described with mean and standard deviation values for continuous variables, absolute numbers, and percentages for categorical variables. The Chi-square test was used to determine the correlation between categorical variables, and the *t*-test between continuous variables. All variables of clinical parameters with a *p* value of <0.05 were considered statistically significant.


**Ethical approval:** The research related to human use has been complied with all the relevant national regulations, institutional policies and in accordance with the tenets of the Helsinki Declaration, and has been approved by the authors’ institutional review board or equivalent committee. The study was approved by the ethics committee of Zhejiang Provincial People’s Hospital and The First People’s Hospital of Aksu Region (No. QT2022349).
**Informed consent:** Because this was a retrospective observational study and had no potential harm to the rights or welfare of subjects, the ethics committee approved the waiver of written informed consent.

## 3 Results

**Figure 2:**
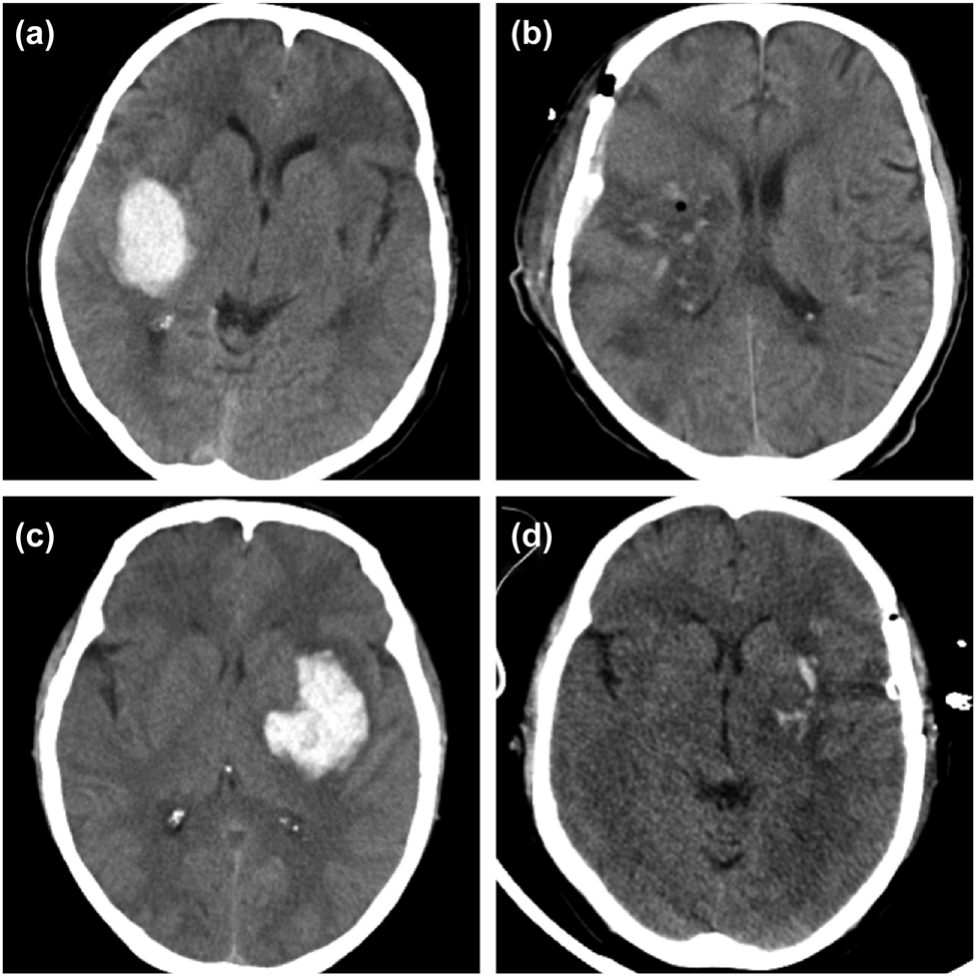
(a and b) Preoperative CT and postoperative CT of case 1 with a right basal ganglia hematoma of 27.4 ml, he received a hematoma evacuation through a transsylvian-transinsular approach 5.2 h from ictus. (c and d) Preoperative CT and postoperative CT representing a left basal ganglia hematoma of 32.8 ml operated through a transsylvian-transinsular approach 4.9 h from ictus.

There was no significant difference in gender, age, hematoma volume, comorbidities, baseline GCS score, and baseline m-NIHSS between the two groups (*p* > 0.05). The mean time from ictus to surgery was 250.3 ± 57.3 min. The mean hematoma clearance rate was 89.7 ± 2.3% (Table 1, Figure 2).

The good recovery proportions of upper and lower limbs in the surgery group were significantly higher than that in the conservative group (*p* < 0.05) at 7 days after hemorrhage. In addition, the good recovery proportion of upper limbs was significantly higher in the surgery group (26 cases, 72.2%) than that in the conservative group (26 cases, 51.0%, *p* < 0.05) at 3 months after hemorrhage (Figure 1). Daily activities using BI scores were significantly higher in the surgery group than in the conservative group (*p* < 0.05) at 3 months after hemorrhage. The favorable prognosis proportion defined as an mRS of 0–3 had no statistically significant difference between the two groups at 3 months after hemorrhage (Table 2).

Patients who had hematoma enlargement or revision surgery were analyzed in the initial groups. Three patients in the conservative group (5.9%) developed hematoma enlargement 6 h after onset, and one of them converted to craniotomy. Of the patients in the surgery group, two (5.6%) had postoperative rebleeding and one of them had revision surgery. Low BI scores between 0 and 20 presented a poor prognosis for the rebleeding cases in both groups (Table 3).

**Table 3:** Complications of treatment in the two groups

	Conservative group (51)	Surgery group (36)
Pulmonary infection	8	7
Tracheotomy	2	0
DVT of lower extremities	3	0
Rebleeding after 6 h/additional surgery	3/1	NA
Postoperative bleeding/Revise surgery	NA	2/1

DVT: deep venous thrombosis; NA: not applicable.

Of the patients in the conservative group, two (3.9%) had a tracheotomy and three (5.9%) had a deep vein thrombosis. The incidences of complications between the two groups had no statistically significant difference (*p* > 0.05, Table 3).

## 4 Discussion

Function recovery is the focal point of moderate basal ganglia hematoma management. Multiple choices including craniotomy with alternative approaches, endoscopic evacuation, stereotaxic drainage, and conservative treatment combined with their timing show the advantages and disadvantages. Although avoiding hemiplegia, aphasia, neglect, and achieving the best motor function recovery is of overwhelming necessity, it is not convenient to make a decision between “Rush to Surgery” and “Wait to Stabilize,” especially considering postoperative rebleeding risk [8]. There are still some subdivisions (such as special surgical approach, the timing of surgery and hematoma volume) worth exploring in the post-STICH era [9]. Therefore, we screened positive measurements from existing surgical options and developed active strategies to achieve satisfactory recovery from basal ganglia hemorrhage.

The motor function is closely related to the pyramidal tracts. Using diffusion-weighted magnetic resonance imaging, Karibe et al. observed the compressive extent of the corticospinal tracts deformed by the hematoma and classified the results into four grades. Grade II was described as the compressive effect that the corticospinal tract was deformed by hematoma compression but not adjacent to hematoma. Surgical treatment should be actively performed to restore motor function in that condition [10]. Ma et al. showed that the FA (fractional anisotropy) value of magnetic resonance imaging of pyramidal tracts decreased within 24 h from ictus, which was closely related to a poor prognosis [11]. Some studies reported that the destruction of the internal capsule was an independent factor of motor function restoration. Internal capsule destruction or compression could be determined by the DTI sequence of MRI, but it was difficult to perform an MRI scan in the present early surgery series. We enrolled the patients with an m-NIHSS of 3 or 4 of motor function, which indicated that the internal capsule was destructed or compressed seriously. Hence, early decompression of the pyramidal tract is helpful for motor recovery in the present study.

Several reports have described that early surgery was beneficial to motor function recovery. Pantazis et al. pointed out that hematoma evacuation within 8 h showed better functional results than conservative treatment [12]. Huang et al. reported that ultra-early stereotactic aspiration within 6 h from the onset of cerebral hemorrhage could effectively improve the neurological function and prognosis [13]. Kaneko et al. reported that 83% of 100 patients with ultra-early craniotomy (within 7 h from ictus) achieved useful functional recovery 6 months after surgery [14]. However, according to the “2020 Chinese Expert Consensus on Endoscopic Surgery for Hypertensive Cerebral Hemorrhage” [1], the endoscopic intervention was not recommended within 6 h from ictus, the benefits of early surgery were difficult to be neglected because of the high incidences and disability rate of basal ganglia hematoma. The literature supports that minimally invasive surgery may be considered to improve functional outcomes, but MIS intends to stabilize ICH before surgery. The optimal time for surgical treatment with MIS remains a controversial issue primarily because of the risk of rebleeding. In the context of ultra-early MIS, it is not strongly recommended in the guidelines of China or 2022 AHA. Several RCTs are underway that will address aspects of these questions.

Craniotomy through the transsylvian-transinsular approach for basal ganglia hematoma evacuation was used for decades and applied in the present study as an active strategy. Several reports have described the benefits of the transsylvian-transinsular approach including better vision, more reliable hemostasis, and acceptable risk of postoperative bleeding. Kim, Xu, Wang, and Gao et al. reported that the transsylvian-transinsular approach could improve long-term prognosis compared with the transcortical approach [2,3,4,5]. The transsylvian-transinsular approach could release cerebrospinal fluid at the beginning of the operation and reduce brain tension. After the Sylvian fissure was opened, there existed only 0.5–1 cm insular cortex thickness above the basal ganglia hematoma, surgeon could employ the keyhole effect to remove the hematoma and decompress the pyramidal tracts. The transsylvian-transinsular approach was also adopted in this study and revealed better recovery of motor function and acceptable risk of postoperative bleeding which was in accordance with the studies aforementioned.

In this study, although mRS at 90 days had no difference between the two groups, the BI score and the good recovery proportion of upper limbs in the surgery group at 90 days were significantly higher simultaneously. The coordination indicated that the BI increase of the surgery group was due to the better motor restoration of the upper limb at 90 days.

For ultra-early surgery, the deepest concern was the risk of postoperative rebleeding which tended to vary considerably in the literature. Some studies showed that the postoperative rebleeding rate did not increase after the removal of moderate hematoma through the transsylvian-transinsular approach. Zhang et al. reported 37 patients underwent hematoma evacuation through the transsylvian-transinsular approach, and no postoperative rebleeding occurred [15]. Wang et al. reported basal ganglia hematoma removal within 6 h after onset, 3 patients (8.5%) in the transsylvian-transinsular group received secondary surgery because of rebleeding and 7 patients (15 %) in the transcortical group [4]. Our study was in accordance with these results and represented a postoperative bleeding rate of 2/36 (5.6%). However, some studies suggested a higher postoperative rebleeding rate associated with early surgery, which mostly applied a transcortical approach. Morgenstern et al. made a cortisectiomy in the middle frontal gyrus and performed the operations within 4 h from onset. The subgroup was terminated unexpectedly due to higher postoperative rebleeding [16]. In 2006, Pantazis et al. conducted a single-center RCT, in which surgery using a transcortical approach was performed to remove the hematoma of 30–80 ml. Hematoma within 3–5 h from onset had a higher postoperative rebleeding rate (22%) while hematoma within 6–8 h with 9% rebleeding rate [12]. Wang et al. reported that the postoperative rebleeding rate increased within 7 h from ictus, and the best time window for surgery was 7–24 h after onset. In his study, the hematoma site was not determined, and only a small part of the treatment was craniotomy [17].

Induction from many previous studies reveals that the approach applied to remove the hematoma plays an important role on the risk of postoperative bleeding as well as the hematoma location and hematoma volume. Transcortical incision to remove deep basal ganglia hematoma forms a longer channel, limits the observation angle, hinders complete hemostasis, and may be related to the increased postoperative rebleeding rate. Comparison of the postoperative rebleeding rate for hematoma volume and surgery approach in various issues are summarized in Table 4.

**Table 4:** Part of previous ultra-early surgical intervention and prognosis studies

Authors and time of publishing	Study classification	Early subgroup population	Ictus to surgery (hour)	Hematoma volume (ml)	Hematoma location	Surgical approach	Postoperative bleeding, *n* (%)
Kaneko et al. [14] (1983)	Case series	100	<7	20–30	Putaminal (lateral type)	Both transsylvian and transtemporal	12/93 (13%)
Morgenstern et al. [16] (2001)	Prospectively	11	<4	23–84	Deep/lobar: 10/1	Cortisectiomy/transcortical	4 (40%)
Mendelow et al. [18] (2005)	RCT, STICH	468	30 (16–49)	40 (24–63)	Lobar, basal ganglia/thalamic, both	Craniotomy (75%), burr hole, endoscopy, stereotaxic	27 (6%)
Pantazis et al. [12] (2006)	Prospective randomized study	54	<8	19 cases (30–80 ml); 35 cases (>80 ml)	Subcortical, putaminal	Transcortical	6 (11%)
Wang et al. [4] (2013)	Respective study	45	<6	42.3 ± 10.1	Basal ganglia/supratentorial hematoma	Transsylvian	3/45
Huang et al. [13] (2021)	Prospective study	47	<6	70.67 ± 5.11	Basal nucleus/lobe: 32/15	Transcortical	1/47 (2.17%)

Our study revealed that the postoperative rebleeding rate was 2/36 (5.6%), which was in accordance with the level of previous studies which applied the transsylvian-transinsular approach. To our experience, the reasonable result was due to the fact that the transsylvian-transinsular approach allowed the surgeon to use a microscope to explore the hematoma cavity and observe the hemostatic effect from multiple angles. The parallel relationship between the Sylvian fissures and the long axes of most basal ganglia hematomas was also beneficial to hematoma evacuation under a microscope. Transcortical (through temporal and frontal lobe) endoscopic approaches could not benefit from the occasion.

Our study excluded large basal ganglia hematoma, integrated the ultra-early timing and the transsylvian-transinsular approach as active strategies, could decompress pyramidal tracts earlier and improve motor recovery, and made it possible for a patient to achieve flexible hands and better completion of daily activities. However, the postoperative m-NIHSS of the surgery group fluctuated which might be due to the operative disturbance, suggesting that the transsylvian-transinsular approach required a meticulous and skillful dissection.

There was no significant difference of postoperative complications between the two groups. The incidence of DVT and tracheotomy were higher in the conservative treatment group, but no significant difference was found.

In terms of limitation, it was a retrospective analysis with a small number of cases in two units. Most follow-ups could not be managed face to face; thus, the Fugl-Meyer motor function score could not be performed during follow-up. The transsylvian-transinsular approach required a meticulous and skillful dissection.

## 5 Conclusion

Ultra-early hematoma evacuation through the transsylvian-transinsular approach are active strategies for moderate basal ganglia hemorrhage, can improve the restoration of motor function, especially of the upper limb, are beneficial to daily activities and long-term prognosis, and are associated with acceptable postoperative complications. These findings suggest that the strategies have the potential for wider application.

## List of abbreviations

STICH: The International Surgical Trial in Intracerebral Hemorrhage
NIHSS: National Institutes of Health Stroke Scale
GCS: Glasgow Coma Scale
mRS: modified Rankin Scale
BI: Barthel Index
CT: Computed tomography
CTA: Computed tomography angiography
IQR: Interquartile range
DVT: Deep vein thrombosis.
